# Efficacy of Traditional Chinese Medicine Injection in Preventing Oxaliplatin-Induced Peripheral Neurotoxicity: An Analysis of Evidence from 3598 Patients

**DOI:** 10.1155/2022/6875253

**Published:** 2022-07-22

**Authors:** Zhi-Ying Chen, Yue Liu, Yuan Wei, Lin-Yao Deng, Qiang Zhang

**Affiliations:** ^1^Department of Pathology, Affiliated Hospital of Chengdu University, Chengdu 610081, Sichuan, China; ^2^Department of Oncology, Jinshan Campus, First Affiliated Hospital of Chongqing Medical University, Chongqing 400016, China; ^3^Xiang Ya Nursing School, Central South University, Changsha 410083, Hunan, China; ^4^Department of Oncology, Army Medical Center of PLA, Chongqing 400042, China

## Abstract

**Background:**

Oxaliplatin is an effective chemotherapeutic agent for the treatment of malignant tumors. However, severe oxaliplatin-induced peripheral neurotoxicity (OIPN) has been well documented. Traditional Chinese medicine injections (TCMIs) have shown significant efficacy in preventing OIPN. However, it is difficult for clinicians to determine the differences in the efficacy of various TCMIs in preventing OIPN. The aim of this study was to compare the efficacy of various TCMIs in preventing OIPN through a network meta-analysis (NMA) to further inform clinical decision-making.

**Methods:**

The Chinese Journal Full Text Database, Chinese Biomedical Literature Database, Wanfang Data Knowledge Service Platform, Chinese Science and Technology Journal Full Text Database, the Cochrane Library, Web of Science, PubMed, and Embase databases were searched for randomized controlled trials (RCTs) of TCMIs for OIPN prevention. The retrieval time was from the establishment of the database to April 12, 2021. NMA was performed using Stata 14.0 software after 2 evaluators independently screened the literature, extracted information, and evaluated the risk of bias of the included studies.

**Results:**

A total of 45 eligible RCTs involving 3598 cancer patients and 13 TCMIs were included. The 13 TCMIs included Xiaoaiping injection (XAPI), compound kushen injection (CKSI), Aidi injection (ADI), Brucea javanica oil emulsion injection (BJOEI), Shenmai injection (SMI), Kangai injection (KAI), *Astragalus* injection (AI), elemene emulsion injection (EEI), Shenfu injection (SFI), Shenqi Fuzheng injection (SIFZI), Kanglaite injection (KLEI), Huachansu injection (HCSI), and lentinan injection (LI). NMA results showed that AI was superior to AD and SIFZI was superior to ADI in reducing the incidence of grade I neurotoxicity. SIFZI was superior to EEI and ADI, and BJOEI was superior to chemotherapy alone in reducing the incidence of grade II neurotoxicity. SMI was superior to LI and CKSI in reducing the incidence of grade III neurotoxicity. SIFZI was superior to LI, BJOEI, XAPI, EEI, SMI, chemotherapy alone, HCSI, KLEI, and ADI in reducing the total incidence of grade I–IV neurotoxicity. SFI was superior to ADI. Based on the SUCRA values, AI was the most likely intervention to reduce the incidence of grade I neurotoxicity, SIFZI was the most likely intervention to reduce the total incidence of grade II and I–IV neurotoxicity, and SMI was the most likely intervention to reduce the incidence of grade III and IV neurotoxicity.

**Conclusion:**

TCMIs can prevent OIPN to some extent, among which SIFZI, SMI, and AI may be the most promising TCMIs. However, given the limitations of current studies, more well-designed, high-quality clinical trials will be needed in the future to validate the benefits of TCMIs.

## 1. Introduction

Oxaliplatin belongs to the third generation of platinum-based antitumor drugs and is the main treatment for many gastrointestinal cancers, especially colorectal cancer [1]. However, up to 40–50% of patients receiving this drug develop oxaliplatin-induced peripheral neurotoxicity (OIPN) [2, 3]. OIPN has a clinically significant impact on the quality of life of patients with cancer and is a dose-limiting toxicity [4, 5]. Up to 90% of patients on oxaliplatin-based regimens with doses ranging from 85 to 130 mg/m^2^ will experience certain degree of acute OIPN [6]. It is characterized by rapid onset of sensory abnormalities and sensory disturbances in the hands, feet, and perioral region, and is essentially reversible within a week [7]. However, about 20–50% of patients develop severe chronic OIPN, and a significant proportion of patients have long-term residual neurotoxicity that severely affects their quality of life [5, 8]. Therefore, how to effectively prevent peripheral neurotoxicity caused by oxaliplatin-containing chemotherapy regimens and mitigate peripheral nervous system injury has become an urgent clinical problem.

At present, there is no specific method for the prevention and treatment of this kind of peripheral neurotoxicity, and symptomatic treatment of Western medicine is mainly used, such as nerve nutrition, nerve growth factor supplementation, and antioxidant treatment with reduced glutathione [9–11]. In fact, the latest oncology guidelines on OIPN acknowledge that despite the large number of trials available, there is no convincing evidence that any interventions are effective in preventing OIPN [12, 13].

OIPN belongs to the category of “paralysis” and “impotence” in Chinese medicine. Many studies have shown that Chinese medicine injections (TCMIs) such as *Astragalus* injection and Shenmai injection have shown good clinical effects in preventing the occurrence of OIPN [14–16]. However, direct comparisons of clinical trials of various TCMIs for OIPN prevention are lacking, and traditional pairwise comparison meta-analyses do not enable comparisons among multiple interventions, making it difficult to assess which intervention has the best efficacy. Compared with traditional pairwise comparison meta-analyses, network meta-analysis (NMA) can not only summarize direct comparative evidence, but also perform indirect comparisons among multiple interventions based on common comparison groups, ranking the efficacy of each intervention, and providing evidence-based medical evidence for clinical drug selection [17, 18]. This study used NMA method to compare the efficacy of TCMIs in OIPN prevention, in order to provide reference for clinical application.

## 2. Methods

NMA was performed in accordance with the Preferred Reporting Items for Systematic Reviews and Meta-Analyses (PRISMA) guidelines [19].

### 2.1. Inclusion and Exclusion Criteria

#### 2.1.1. Types of Studies

Randomized controlled trials(RCTs) were included.

#### 2.1.2. Participants

Patients with a diagnosis of malignancy confirmed by histopathology and/or cytology or imaging. Treatment with oxaliplatin or oxaliplatin-containing chemotherapy regimens was specified in the chemotherapy regimen.

#### 2.1.3. Interventions and Comparisons

The control group was given chemotherapy alone or chemotherapy with placebo. The experimental group used TCMIs in addition to chemotherapy, and the type, dose, and frequency of TCMIs were not limited.

#### 2.1.4. Outcomes

The incidence of OIPN includes the incidence of grade I neurotoxicity, grade II neurotoxicity, grade III neurotoxicity, grade IV neurotoxicity, and the total incidence of grade I–IV neurotoxicity.

### 2.2. Exclusion Criteria

- Republished literature
- Literature with incomplete data
- Both groups used other Chinese medical treatments such as traditional Chinese medicine decoction, Chinese patent medicine, or acupuncture
- Nonrandomized controlled trial

### 2.3. Search Strategy

The Chinese Journal Full Text Database, Chinese Biomedical Literature Database, Wanfang Data Knowledge Service Platform, Chinese Science and Technology Journal Full Text Database, the Cochrane Library, Web of Science, PubMed, and Embase databases were searched for randomized controlled trials (RCTs) of TCMIs for OIPN prevention. The retrieval time was from the establishment of the database to April 12, 2021. Search terms included oxaliplatin, neurotoxicity, names of included TCMIs, RCTs, and their synonyms. The search strategy was developed according to the criteria of the Cochrane systematic review handbook. Taking PubMed database as an example, detailed search strategies are shown in supplementary materials (Table S1).

### 2.4. Data Extraction

Two researchers independently screened the literature, extracted information, and cross-checked it according to inclusion and exclusion criteria. In case of any disagreement, it was resolved through discussion or referred to a third party for negotiation. The data extraction included (1) basic information of the included studies, including study title, first author, journal of publication, and time; (2) baseline characteristics of the study population, including sample size of each group, age of patients, population origin, and tumor type; (3) specific details of the interventions, including the TCMIs used and the type of chemotherapeutic agents; (4) key elements of bias risk assessment; and (5) outcome indicators and outcome measures of interest, including the measurement tools for OIPN and the incidence of OIPN.

### 2.5. Quality Assessment

The risk of bias for RCTs was evaluated by 2 investigators according to the Cochrane systematic review handbook [20]. Evaluation elements included randomization method, concealment of grouping scheme, blinding, completeness of outcome data, selective reporting of study results, and other sources of bias. These elements were assessed as “low risk,” “high risk,” and “unclear.”

### 2.6. Data Analysis

Count data were analyzed with relative risk (RR) and 95% confidence interval (95% CI) as efficacy statistics. *I*^2^ was used to quantitatively determine the magnitude of heterogeneity. If *I*^2^ < 50% and *P* > 0.1, meta-analysis was performed using a fixed-effects model. If *I*^2^ ≥ 50% and *P* < 0.1, meta-analysis was performed using a random-effects model. Since this study was an indirect comparison of various TCMIs combined with chemotherapy based on chemotherapy, no consistency test was required. Network group commands were used for data preprocessing in NMA. Network evidence plots and “corrected-comparison” funnel plots were drawn for each outcome indicator, and pairwise comparisons of different interventions were performed. Efficacy was ranked according to the surface under the cumulative ranking curve (SUCRA). Stata 14.0 was used for direct comparison meta-analysis, NMA, and graph drawing.

## 3. Results

### 3.1. Literature Screening Result

**Figure 1:**
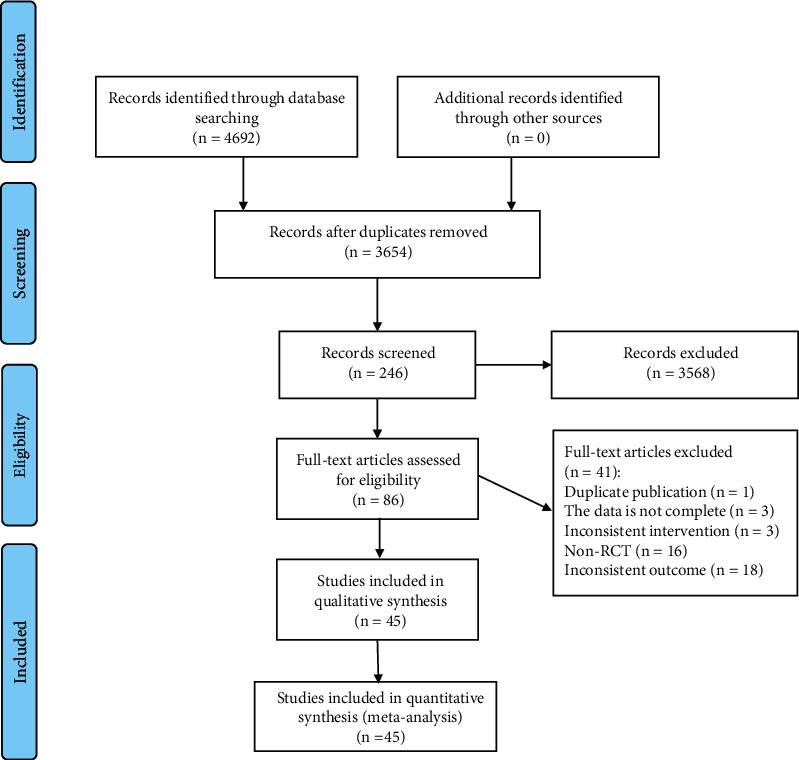
Flowchart of the literature screening process.

A total of 4692 literature were retrieved through electronic databases, and 1038 duplicates were removed. 3568 literature were excluded by reading the titles and abstracts. The remaining 246 literature were read through the full text, and finally 45 literature [14–16, 21–62] were included. The literature selection process is illustrated in Figure 1.

### 3.2. Basic Characteristics of Included Studies

The 45 RCTs [14–16, 21–49, 51–62] were included in two-arm trials, including 3598 patients with cancer. A total of 13 TCMIs were included, including Xiaoaiping injection (4 items) [34, 42, 45, 48], *Astragalus* injection (3 items) [14, 16, 30], Aidi injection (10 items) [23, 26, 27, 33, 39, 46, 48, 53, 56, 57], Brucea javanica oil emulsion injection (3 items) [22, 40, 50], compound kushen injection (8 items) [25, 35–37, 51, 54, 55, 59], elemene emulsion injection (1 items) [24], Huachansu injection (2 items) [28, 49], Kangai injection (3 items) [21, 29, 31], Kanglaite injection (1 items) [43], lentinan injection (1 items) [38], Shenfu injection (3 items) [32, 47, 62], Shenmai injection (4 items) [15, 58, 60, 61], and Shenqi Fuzheng injection (2 items) [44, 52]. All trials were conducted in China. The included tumor types were basically gastric and colorectal cancers. The measurement tools of OIPN included WHO classification criteria for acute and subacute toxicity of anticancer drugs, the National Cancer Institute Common Terminology Criteria for Adverse Events, and Oxaliplatin Levi-specific sensory neurotoxicity grading. The details of the study characteristics are depicted in Table 1.

### 3.3. Risk of Bias Assessment

**Figure 2:**
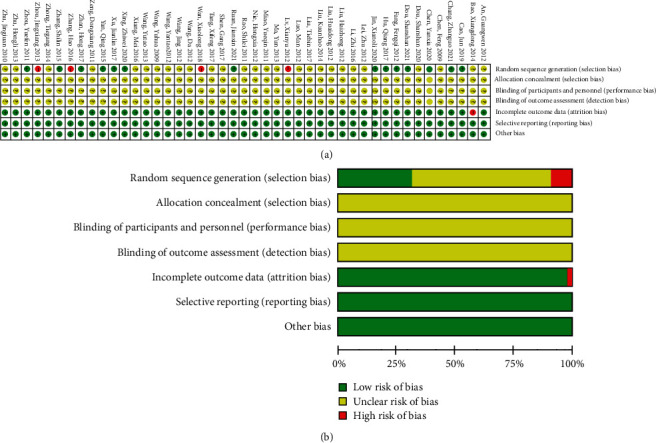
Risk of bias graph of the included RCTs. (a) Risk of bias summary; (b) risk of bias graph.

14 studies were considered low risk for randomization, 4 studies were assessed as high risk because they had incorrect methods of random sequence generation, and the randomization of the remaining 27 studies was unclear. The method of allocation concealment was unclear for all studies. Due to the specificity of TCMIs, it is difficult to do blinding. The blinding method for all studies was unclear. For incomplete outcome data, one study showed high risk of bias. Details of the risk of bias assessment are shown in Figure 2.

### 3.4. Directly Compared Meta-Analysis Results

A meta-analysis of direct comparisons of TCMIs combined with chemotherapy compared to chemotherapy alone was conducted. The results showed that AI can reduce incidence of grade I neurotoxicity (*P* < 0.05) compared with chemotherapy; SFI and AI could reduce incidence of grade II neurotoxicity (*P* < 0.05); SFI and SMI could reduce incidence of grade III neurotoxicity (*P* < 0.05); ADI, SFI, SMI, SIFZI, CKSI, HCSI, AI, KLEI, LI, and XAPI could reduce total incidence of grade I ∼ IV neurotoxicity (*P* < 0.05). Results of direct comparative meta-analyses are shown in Table 2.

### 3.5. Comparison Results of Network Meta-Analysis

#### 3.5.1. Evidence Network Diagram

**Figure 3:**
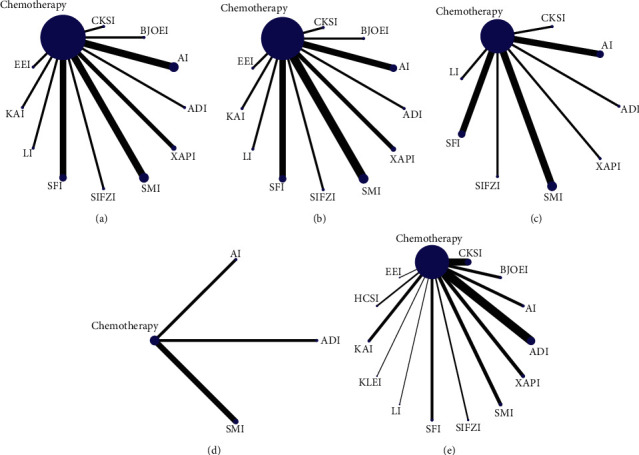
Network diagrams for different outcomes. (a) Incidence of grade I neurotoxicity; (b) incidence of grade II neurotoxicity; (c) incidence of grade III neurotoxicity; (d) incidence of grade IV neurotoxicity; (e) total incidence of grade I ∼ IV neurotoxicity.

The evidence network diagram is illustrated in Figure 3. Each dot represents a drug, and the direct connection between the two points indicated a direct comparison between the two drugs. The thicker the line between the two dots, the greater the number of paired studies, the larger the node, and the larger the sample size of studies involved in the intervention.

#### 3.5.2. Incidence of Grade I Neurotoxicity

**Figure 4:**
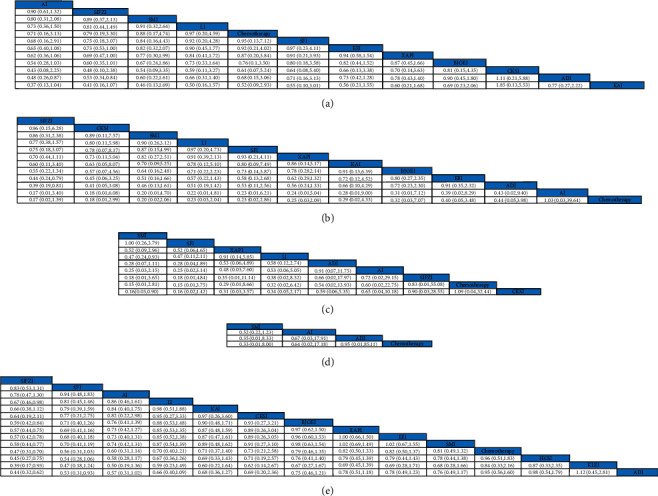
Pooled estimates of the network meta-analysis. (a) Pooled relative risk (95% confidence intervals) for the incidence of grade I neurotoxicity; (b) pooled relative risk (95% confidence intervals) for the incidence of grade II neurotoxicity; (c) pooled relative risk (95% confidence intervals) for the incidence of grade III neurotoxicity; (d) pooled relative risk (95% confidence intervals) for the incidence of grade IV neurotoxicity; (e) pooled relative risk (95% confidence intervals) for the total incidence of grade I ∼ IV neurotoxicity.

Incidence of grade I neurotoxicity was reported in 20 studies involving 11 TCMIs and 1522 patients. The results of the NMA showed that the differences were statistically significant for AI versus ADI (RR:0.48; 95%CI (0.26, 0.87)), and SIFZI versus ADI (RR:0.53; 95%CI (0.34, 0.84)), and there were no significant differences in other interventions (Figure 4(a)).

#### 3.5.3. Incidence of Grade II Neurotoxicity

Incidence of grade II neurotoxicity was reported in 19 studies involving 11 TCMIs and 1462 patients. The results of the NMA showed statistically significant differences for SIFZI versus EEI (RR:0.44; 95%CI (0.24, 0.79)), SIFZI versus ADI (RR:0.39; 95%CI (0.19, 0.81)), and BJOEI versus chemotherapy alone (RR:0.32; 95%CI (0.03, 3.07)), and the difference between the remaining interventions was not statistically significant (Figure 4(b)).

#### 3.5.4. Incidence of Grade III Neurotoxicity

Incidence of grade III neurotoxicity was reported in 15 studies involving 8 TCMIs and 1227 patients. The results of the NMA showed statistically significant differences in SMI versus LI (RR:0.47; 95%CI (0.24, 0.93)), and SMI versus CKSI (RR:0.16; 95%CI (0.03, 0.90)), and the difference between the remaining interventions was not statistically significant (Figure 4(c)).

#### 3.5.5. Incidence of Grade IV Neurotoxicity

Incidence of grade IV neurotoxicity was reported in 4 studies involving 3 TCMIs and 355 patients. The results of the NMA showed no statistically significant differences in the comparison of the interventions (Figure 4(d)).

#### 3.5.6. Total Incidence of Grade I ∼ IV Neurotoxicity

Total incidence of grade I ∼ IV neurotoxicity was reported in 45 studies involving 13 TCMIs and 3598 patients. The results of the NMA showed that the differences were statistically significant for SIFZI versus LI (RR:0.67; 95%CI (0.46, 0.98)), SIFZI versus BJOEI (RR:0.59; 95%CI (0.42, 0.84)), SIFZI versus XAPI (RR:0.57; 95%CI (0.44, 0.75)), SIFZI versus EEI (RR:0.57; 95%CI (0.42, 0.78)), SIFZI versus SMI (RR:0.58; 95%CI (0.44, 0.77)), SIFZI versus chemotherapy (RR:0.47; 95%CI (0.31, 0.70)), SIFZI versus HCSI (RR:0.45; 95%CI (0.27, 0.75)), SIFZI versus KLEI (RR:0.39; 95%CI (0.17, 0.93)), SIFZI versus ADI (RR:0.44; 95%CI (0.32, 0.62)), and SFI versus ADI (RR:0.53; 95%CI (0.31, 0.93)), and the differences between the remaining interventions were not statistically significant (Figure 4(e)).

### 3.6. Rank Probabilities

**Figure 5:**
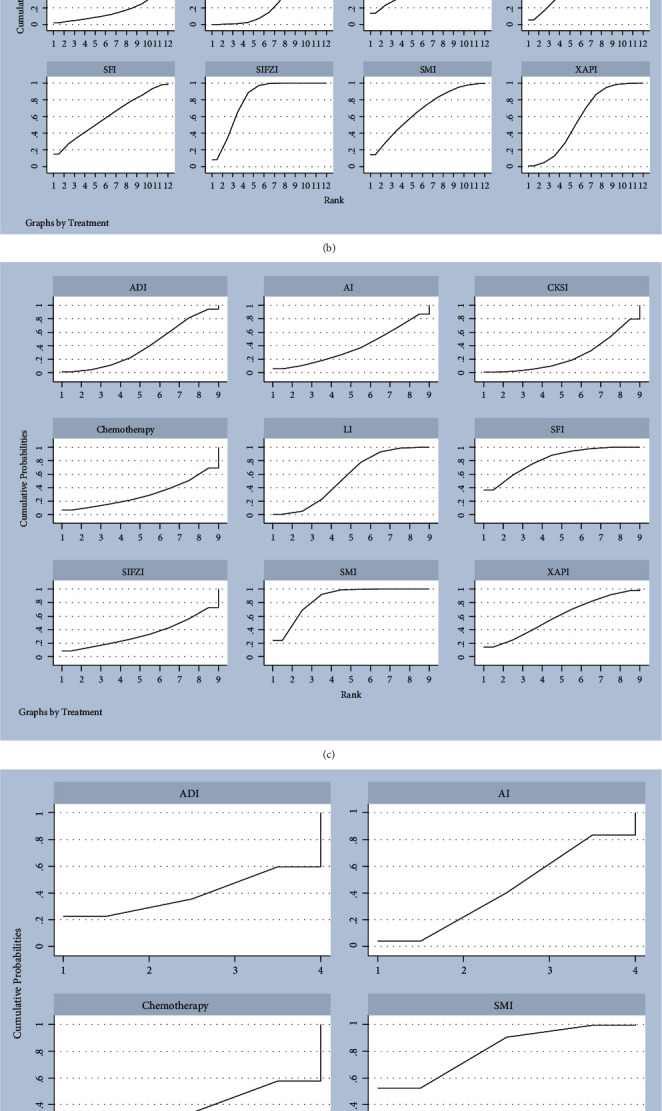
The surface under the cumulative ranking curve (SUCRA) plots for different outcomes. (a) Incidence of grade I neurotoxicity; (b) incidence of grade II neurotoxicity; (c) incidence of grade III neurotoxicity; (d) incidence of grade IV neurotoxicity; (e) total incidence of grade I ∼ IV neurotoxicity.

The SUCRA cumulative probability ranking showed that AI was most likely to be the best intervention to reduce the incidence of grade I neurotoxicity. Ranking results of incidence of grade I neurotoxicity were AI (SUCRA = 84.1%) > SIFZI (SUCRA = 78.4%) > SMI (SUCRA = 64.2%) > LI (SUCRA = 58.5%) > chemotherapy (SUCRA = 54.5%)>SFI (SUCRA = 52.5%)>EEI (SUCRA = 49.7%)>XAPI (SUCRA = 44.8)>BJOEI (SUCRA = 34.6%)>CKSI (SUCRA = 33.7%)>ADI (SUCRA = 26.4%)>KAI (SUCRA = 18.6%) (Figure 5(a)). SIFZI was the most likely intervention to reduce the incidence of grade II neurotoxicity. Ranking results of incidence of grade II neurotoxicity were SIFZI (SUCRA = 81.2%) > CKSI (SUCRA = 68.5%) > SMI (SUCRA = 67.8%) > LI (SUCRA = 63.6%) > SFI (SUCRA = 60.8%) > XAPI (SUCRA = 58.8%) > KAI (SUCRA = 51.9%) > BJOEI (SUCRA = 45.1%) > EEI (SUCRA = 32.3%) > ADI (SUCRA = 29.8%) > AI (SUCRA = 22.8%) > chemotherapy (SUCRA = 17.4%) (Figure 5(b)). SMI was the most likely intervention to reduce the incidence of grade III neurotoxicity. Ranking results of incidence of grade III neurotoxicity were SMI (SUCRA = 85.6%) > SFI (SUCRA = 81.2%) > XAPI (SUCRA = 59.5%) > LI (SUCRA = 55.9%) > ADI (SUCRA = 39.5%) > AI (SUCRA = 38.3%) > SIFZI (SUCRA = 34.2%) > chemotherapy (SUCRA = 30.4%)>CKSI (SUCRA = 25.4%) (Figure 5(c)). SMI was the most likely intervention to reduce the incidence of grade IV neurotoxicity. Ranking results of incidence of grade IV neurotoxicity were SMI (SUCRA = 80.7%) > AI (SUCRA = 42.5%) > ADI (SUCRA = 39.2%) > chemotherapy (SUCRA = 37.6%) (Figure 5(d)). SIFZI was the most likely intervention to reduce the incidence of grade I ∼ IV neurotoxicity. Ranking results of incidence of grade I ∼ IV neurotoxicity were SIFZI (SUCRA = 94.5%) > SFI (SUCRA = 79.4%) > AI (SUCRA = 75.0%) > LI (SUCRA = 62.0%) > KAI (SUCRA = 59.0%) > CKSI (SUCRA = 53.1%) > BJOEI (SUCRA = 50.2%) > XAPI (SUCRA = 45.5%) > EEI (SUCRA = 45.0%) > SMI (SUCRA = 47.7%) > chemotherapy (SUCRA = 24.6%)>HCSI (SUCRA = 23.4%)>KLEI (SUCRA = 21.2%)>ADI (SUCRA = 19.2%) (Figure 5(e)).

### 3.7. Small-Sample Effect Estimation

**Figure 6:**
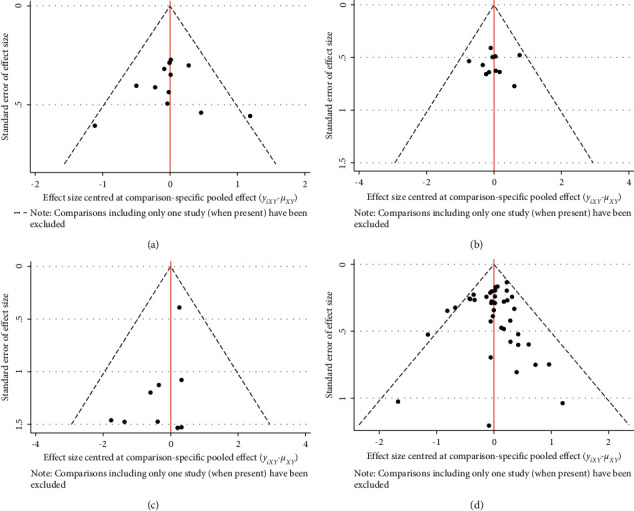
Publication bias. (a) Incidence of grade I neurotoxicity comparison-correction funnel chart; (b) incidence of grade II neurotoxicity comparison-correction funnel chart; (c) incidence of grade III neurotoxicity comparison-correction funnel chart; (d) total incidence of grade I ∼ IV neurotoxicity comparison-correction funnel chart.

If no less than 10 studies were included, comparison-corrected funnel plots were drawn to identify the possibility of small-sample effects in the intervention network. The resulting funnel plot was slightly asymmetric, considering the possibility of a small-sample effect or publication bias between studies (Figure 6).

### 3.8. Sensitivity Analysis

We performed sensitivity analyses for outcome indicators that included at least 3 or more literature. Sensitivity analysis showed that SMI plus chemotherapy versus chemotherapy reversed the results of the meta-analysis in terms of incidence of grade I neurotoxicity and incidence of grade II neurotoxicity. The results of the meta-analysis were reversed for AI plus chemotherapy versus chemotherapy in terms of incidence of grade II neurotoxicity. KAI plus chemotherapy versus chemotherapy and XAPI plus chemotherapy versus chemotherapy were reversed for incidence of grade I ∼ IV neurotoxicity. The results of the meta-analysis were reversed for neurotoxicity. No reversal was found for the remaining outcome indicators. The results are presented in the Supplementary Material (Figures S1–S17).

## 4. Discussion

OIPN is the primary dose-limiting toxicity of oxaliplatin and is characterized by specific somatosensory features, including cold and mechanical abnormal pain [63]. The pathogenesis of OIPN is still unclear, and there are several theories of its pathogenesis: ion channel theory, axonal neuropathy theory, central neuro-sensitive theory, neuronal cell death theory, etc. [64–67]. The main therapeutic drugs in Western medicine are sodium channel blockers, calcium-magnesium combination, reduced glutathione, gangliosides, and venlafaxine [68–73]. However, based on the current evidence, particularly the results of an NMA, there is insufficient certainty to support that any Western drug is effective in preventing OIPN [4]. TCMIs are the product of modernization of Chinese medicine, and compared with other herbal dosage forms, the injectable form has the characteristics of high bioavailability, precise efficacy, and rapid action, and is mostly used for preventive treatment in clinical practice. In this study, we performed NMA on 13 TCMIs and combined the results to determine which TCMIs are the best choice for clinical treatment and to provide reference for clinicians to prevent the occurrence of OIPN.

The NMA evaluated the efficacy of 13 TCMIs for the prevention of OIPN in 3598 cancer patients. 13 TCMIs include XAPI, CKSI, ADI, BJOEI, SMI, KAI, AI, EEI, SFI, SIFZI, KLEI, HCSI, and LI. The NMA results showed that AI was better than ADI and SIFZI was better than ADI in preventing the incidence of grade I neurotoxicity, and the probability ranking showed that AI > SIFZI > SMI > LI > chemotherapy > SFI > EEI > XAPI > BJOEI > CKSI > ADI > KAI. SIFZI was superior to EEI and ADI, and BJOEI was superior to chemotherapy alone in preventing the incidence of grade II neurotoxicity. The probability ranking results showed that SIFZI > CKSI > SMI > LI > SFI > XAPI > KAI > BJOEI > EEI > ADI > AI > chemotherapy. SMI was superior to LI and CKSI in preventing the incidence of grade III neurotoxicity. Probability ranking results show that SMI > SFI > XAPI > LI > ADI > AI > SIFZI > chemotherapy > CKSI. There was no statistically significant difference between the interventions in preventing the incidence of grade IV neurotoxicity. The probability ranking results showed that SMI > AI > ADI > chemotherapy. SIFZI was superior to LI, BJOEI, XAPI, EEI, SMI, chemotherapy alone, HCSI, KLEI, and ADI in preventing grade I ∼ IV neurotoxicity; SFI was superior to ADI. The probability ranking results showed that SIFZI > SFI > AI > LI > KAI > CKSI > BJOEI > XAPI > EEI > SMI > chemotherapy > HCSI > KLEI > ADI. SIFZI, SMI, and AI had the largest SUCRA values and were most likely to be the best treatment options. Considering the moderate quality of the included studies and the limited number of included studies, the probability ranking results are for clinicians' reference only.

In vitro and in vivo studies suggest that extracts of *Astragalus* may be a potential nerve growth-promoting factor that helps promote the growth of peripheral nerve axons [74]. Astragaloside IV, an active component of *Astragalus*, attenuates OIPN by modulating neuroinflammation and oxidative stress and downregulating the expression of TNF-*α*, IL-6, and IL-1*β* [75, 76]. A study by Luo et al. [16] showed that AI reduced the overall incidence of OIPN. In addition, the results of a meta-analysis showed that Astragalus-based herbal medicines helped to alleviate OIPN [77]. Therefore, Astragalus-based TCMIs (e.g., AI and SIFZI) may be a direction for future research. However, based on the probability ranking results, AI is the best intervention to reduce the incidence of grade I neurotoxicity and SIFZI is most likely to be the best intervention to reduce the total incidence of grade II and I ∼ IV neurotoxicity. This may be because codonopsis can regulate immunity, increase bone marrow hematopoiesis, inhibit platelet aggregation, improve microcirculation of surrounding tissues, and protect nerve function [44]. From the perspective of the theory of traditional Chinese medicine, if qi and blood are not running smoothly, the skin will be numb if it is not nourishing, and SIFZI has the effect of nourishing qi to support the righteousness. SMI is purified from ginseng and Ophiopogon japonicus. It contains ginsenosides, which can regulate the metabolism of neurons and promote the repair of damaged neurons [15, 78]. Ophiopogon japonicus is a natural antioxidant agent, which can directly reduce the production of oxygen-free radicals, reduce the lipid peroxidation of cells, and enhance the antioxidant function of the body [79]. The meta-analysis of direct comparisons in this study found that SMI reduced the total incidence of grade I ∼ IV neurotoxicity. Probability ranking results suggest that SMI is most likely to be the best intervention for reducing the incidence of grade III and IV neurotoxicity. SIFZI, SMI, and AI may be the most promising TCMIs in preventing the occurrence of OIPN.

In this study, NMA was used for the first time to compare the clinical efficacy of different TCMIs in the prevention of OIPN, with a large number of included studies and a large sample size, showing high statistical efficacy. However, there were also certain limitations: (i) the included studies were all in Chinese, which may have language bias; (ii) the quality of the included studies was average, and most of them did not mention allocation concealment and blinding, which may affect the reliability of the results; (iii) there was some heterogeneity in some results, which may be related to the clinical characteristics of the included studies such as different tumor types and chemotherapy regimens. (iv) The included RCTs were compared on the basis of chemotherapy combined with TCMIs and chemotherapy alone, and there was a lack of direct comparison between TCMI. This may have weakened the strength of the evidence supporting the results. Therefore, future high-quality randomized controlled trials are needed to assess the clinical efficacy of TCMIs for the prevention of OIPN.

In summary, the application of TCMIs on top of oxaliplatin-containing chemotherapy regimens can prevent the occurrence of OIPN to some extent. Among them, AI focused on reducing grade I neurotoxic reactions, SIFZI focused on reducing grade II and I ∼ IV neurotoxic reactions, and SMI focused on reducing grade III and IV neurotoxic reactions. However, based on the limitations of this study, the efficacy ranking does not fully indicate the clinical efficacy, and the results of this ranking should be viewed with caution.

## Figures and Tables

**Table 1:** Characteristics of included studies.

Study	Stata	Sample size	Age		Traditional Chinese medicine injection	Chemotherapy drugs	Tumor type	No. of patients (start)	No. of patients (end)	Neurotoxicity assessment tool
		Experimental group/control group	Experimental group	Control group						
Wang et al. [45]	China	46/36	32∼74		Xiaoaiping injection	Oxaliplatin + capecitabine tablets	Colorectal cancer	82	82	WHO classification criteria for acute and subacute toxicity of anticancer drugs
Liu et al. [35]	China	77/75	58 ± 2.4	61 ± 1.3	Compound kushen injection	Oxaliplatin+5-fluorouracil + calcium folinate	Gastric cancer	152	152	WHO classification criteria for acute and subacute toxicity of anticancer drugs
Liu et al. [33]	China	28/28	42∼75	35∼76	Aidi injection	Oxaliplatin + tegafur-gimeracil-oteracil potassium capsule	Gastric cancer	56	56	WHO classification criteria for acute and subacute toxicity of anticancer drugs
Liu et al. [34]	China	28/28	28∼70		Xiaoaiping injection	Oxaliplatin+5-fluorouracil + calcium folinate	Gastric cancer	56	56	The National Cancer Institute Common Terminology Criteria for Adverse Events
Liu et al. [36]	China	68/68	61.4 ± 12.3	61.8 ± 12.6	Compound kushen injection	Oxaliplatin+5-fluorouracil + calcium folinate	Gastric cancer	136	136	WHO classification criteria for acute and subacute toxicity of anticancer drugs
Bao et al. [22]	China	63/62	62.0 ± 9.4	63.0 ± 7.5	Brucea javanica oil emulsion injection	Oxaliplatin+5-fluorouracil + calcium folinate	Colorectal cancer	130	125	The National Cancer Institute Common Terminology Criteria for Adverse Events
Zhan et al. [55]	China	64/64	62.1 ± 11.6	62.4 ± 11.8	Compound kushen injection	Oxaliplatin+5-fluorouracil + Calcium folinate	Colorectal cancer	128	128	WHO classification criteria for acute and subacute toxicity of anticancer drugs
Xiang et al. [51]	China	39/39	39.8 ± 7.3	43.7 ± 6.2	Compound kushen injection	Raltitrexed + oxaliplatin	Colorectal cancer	78	78	WHO classification criteria for acute and subacute toxicity of anticancer drugs
Lv et al. [37]	China	33/30	58	61	Compound kushen injection	Oxaliplatin+5-fluorouracil + calcium folinate	Gastric cancer	63	63	WHO classification criteria for acute and subacute toxicity of anticancer drugs
Zhou et al. [59]	China	41/43	53.8 ± 8.1	58.3 ± 5.2	Compound kushen injection	Oxaliplatin+5-fluorouracil + calcium folinate	Colorectal cancer	84	84	WHO classification criteria for acute and subacute toxicity of anticancer drugs
Zhou et al. [60]	China	40/40	28∼67		Shenmai injection	Oxaliplatin	Gastric cancer, Colorectal cancer	80	75	Oxaliplatin Levi-specific sensory neurotoxicity grading
An et al. [21]	China	38/32	45∼75	44∼71	Kangai injection	Oxaliplatin+5-fluorouracil + calcium folinate	Gastric cancer	70	70	WHO classification criteria for acute and subacute toxicity of anticancer drugs
Chang et al. [25]	China	53/53	63.74 ± 7.85	64.63 ± 8.25	Compound kushen injection	Oxaliplatin + tegafur-gimeracil-oteracil potassium capsule	Gastric cancer	106	106	WHO classification criteria for acute and subacute toxicity of anticancer drugs
Zhang et al. [57]	China	50/50	56.7 ± 5.3	55.6 ± 3.4	Aidi injection	Oxaliplatin + tegafur-gimeracil-oteracil potassium capsule	Colorectal cancer	100	100	WHO classification criteria for acute and subacute toxicity of anticancer drugs
Zhang et al. [56]	China	43/43	61.9 ± 3.9	62.1 ± 3.9	Aidi injection	Oxaliplatin+5-fluorouracil + calcium folinate	Colorectal cancer	86	86	WHO classification criteria for acute and subacute toxicity of anticancer drugs
Xu et al. [53]	China	47/47	53.42 ± 3.96	54.29 ± 4.11	Aidi injection	Oxaliplatin + tegafur-gimeracil-oteracil potassium capsule	Gastric cancer	94	94	WHO classification criteria for acute and subacute toxicity of anticancer drugs
Fang et al. [15]	China	46/50	54∼80	49∼78	Shenmai injection	Oxaliplatin+5-fluorouracil + calcium folinate	Gastric cancer, colorectal cancer	96	96	Oxaliplatin Levi-specific sensory neurotoxicity grading
Jin et al. [30]	China	52/52	58.12 ± 7.69	58.34 ± 7.25	*Astragalus* injection	Oxaliplatin+5-fluorouracil + calcium folinate	Colorectal cancer	104	104	WHO classification criteria for acute and subacute toxicity of anticancer drugs
Cao et al. [23]	China	35/35	48.51 ± 11.85	48.4 ± 11.85	Aidi injection	Oxaliplatin+5-fluorouracil + Calcium folinate	Gastric cancer	70	70	WHO classification criteria for acute and subacute toxicity of anticancer drugs
Zeng et al. [24]	China	25/24	31∼75	32∼74	Elemene emulsion injection	Oxaliplatin+5-fluorouracil + calcium folinate	Gastric cancer	49	49	WHO classification criteria for acute and subacute toxicity of anticancer drugs
Zhu et al. [61]	China	46/41	29∼73		Shenmai injection	Oxaliplatin+5-fluorouracil + calcium folinate	Colorectal cancer	87	87	Oxaliplatin Levi-specific sensory neurotoxicity grading
Zhu et al. [62]	China	40/40	52.1	50.8	Shenfu injection	Oxaliplatin+5-fluorouracil + calcium folinate, oxaliplatin + capecitabine tablets	Gastric cancer, colorectal cancer	80	80	Oxaliplatin Levi-specific sensory neurotoxicity grading
Li et al. [32]	China	40/40	32∼76	31∼75	Shenfu injection	Oxaliplatin+5-fluorouracil + calcium folinate	Gastric cancer, colorectal cancer	80	80	Oxaliplatin Levi-specific sensory neurotoxicity grading
Tang et al. [44]	China	31/30	60.69 ± 3.13	59.16 ± 3.15	Shenqi Fuzheng injection	Oxaliplatin + tegafur-gimeracil-oteracil potassium capsule	Gastric cancer	61	61	WHO classification criteria for acute and subacute toxicity of anticancer drugs
Shen et al. [43]	China	54/50	31∼75		Kanglaite injection	Oxaliplatin + tegafur-gimeracil-oteracil potassium capsule	Gastric cancer	104	104	WHO classification criteria for acute and subacute toxicity of anticancer drugs
Wang et al. [48]	China	38/36	32∼74		Aidi injection	Oxaliplatin+5-fluorouracil + calcium folinate	Colorectal cancer	74	74	WHO classification criteria for acute and subacute toxicity of anticancer drugs
Wang et al. [49]	China	36/32	40∼72		Huachansu injection	Oxaliplatin+5-fluorouracil + calcium folinate	Gastric cancer	68	68	WHO classification criteria for acute and subacute toxicity of anticancer drugs
Wang et al. [50]	China	24/23	31∼75	32∼74	Brucea javanica oil emulsion injection	Oxaliplatin+5-fluorouracil + calcium folinate	Gastric cancer	47	47	WHO classification criteria for acute and subacute toxicity of anticancer drugs
Wang et al. [46]	China	32/31	61.2 ± 3.8	62.1 ± 3.5	Aidi injection	Oxaliplatin+5-fluorouracil + calcium folinate	Colorectal cancer	63	63	WHO classification criteria for acute and subacute toxicity of anticancer drugs
Wang et al. [47]	China	40/40	51.4	51.4	Shenfu injection	Oxaliplatin+5-fluorouracil + calcium folinate, oxaliplatin + capecitabine tablets	Gastric cancer, colorectal cancer	80	80	WHO classification criteria for acute and subacute toxicity of anticancer drugs
Dou et al. [27]	China	34/34	58.4 ± 12.9	59.2 ± 18.3	Aidi injection	Oxaliplatin + tegafur-gimeracil-oteracil potassium capsule	Colorectal cancer	68	68	WHO classification criteria for acute and subacute toxicity of anticancer drugs
Dou et al. [28]	China	36/36	57.0 ± 3.2	56.3 ± 2.4	Huachansu injection	Oxaliplatin + tegafur-gimeracil-oteracil potassium capsule	Gastric cancer	72	72	WHO classification criteria for acute and subacute toxicity of anticancer drugs
Miao et al. [39]	China	41/43	65		Aidi injection	Oxaliplatin+5-fluorouracil + calcium folinate	Gastric cancer	84	84	WHO classification criteria for acute and subacute toxicity of anticancer drugs
Nie et al. [40]	China	30/30	30∼73	27∼75	Brucea javanica oil emulsion injection	Oxaliplatin+5-fluorouracil + calcium folinate	Colorectal cancer	60	60	WHO classification criteria for acute and subacute toxicity of anticancer drugs
Hu et al. [29]	China	18/18	50∼80	60∼85	Kangai injection	Oxaliplatin + tegafur-gimeracil-oteracil potassium capsule	Gastric cancer	36	36	The National Cancer Institute Common Terminology Criteria for Adverse Events
Xing et al. [52]	China	45/45	54.1 ± 8.4	53.3 ± 8.5	Shenqi Fuzheng injection	Oxaliplatin+5-fluorouracil + calcium folinate	Colorectal cancer	90	90	WHO classification criteria for acute and subacute toxicity of anticancer drugs
Zhong et al. [58]	China	30/30	62.5 ± 10.7	60.5 ± 9.2	Shenmai injection	Oxaliplatin+5-fluorouracil + calcium folinate	Gastric cancer, duodenal carcinoma, colorectal cancer	60	60	WHO classification criteria for acute and subacute toxicity of anticancer drugs
Yan et al. [54]	China	41/41	55.1 ± 6.8	53.6 ± 6.1	Compound kushen injection	Oxaliplatin+5-fluorouracil + calcium folinate	Colorectal cancer	82	82	WHO classification criteria for acute and subacute toxicity of anticancer drugs
Ruan et al. [42]	China	42/42	36∼74	38∼76	Xiaoaiping injection	Oxaliplatin + tegafur-gimeracil-oteracil potassium capsule	Gastric cancer	84	84	The National Cancer Institute Common Terminology Criteria for Adverse Events
Chen et al. [14]	China	30/30	53.8 ± 14.4	52.5 ± 12.6	*Astragalus* injection	Oxaliplatin+5-fluorouracil + calcium folinate	Colorectal cancer	60	60	WHO classification criteria for acute and subacute toxicity of anticancer drugs
Chen et al. [26]	China	45/45	46.93 ± 6.91	46.77 ± 6.83	Aidi injection	Oxaliplatin + tegafur-gimeracil-oteracil potassium capsule	Gastric cancer	90	90	WHO classification criteria for acute and subacute toxicity of anticancer drugs
Lei et al. [31]	China	30/30	31∼75		Kangai injection	Oxaliplatin+5-fluorouracil + calcium folinate	Colorectal cancer	60	60	WHO classification criteria for acute and subacute toxicity of anticancer drugs
Rao et al. [41]	China	30/30	31∼74	34∼72	Xiaoaiping injection	Oxaliplatin+5-fluorouracil + calcium folinate	Colorectal cancer	60	60	WHO classification criteria for acute and subacute toxicity of anticancer drugs
Ma et al. [38]	China	41/37	61.6 ± 8.19	63.41 ± 7.43	Lentinan injection	Oxaliplatin+5-fluorouracil + calcium folinate	Colorectal cancer	78	78	WHO classification criteria for acute and subacute toxicity of anticancer drugs
Luo et al. [16]	China	30/30	46∼73	42∼74	*Astragalus* injection	Oxaliplatin+5-fluorouracil + calcium folinate, oxaliplatin + capecitabine tablets	Colorectal cancer	60	60	WHO classification criteria for acute and subacute toxicity of anticancer drugs

**Table 2:** Direct comparison of meta-analysis results.

Outcome index	Comparison category	Number of studies	Heterogeneity		Meta-analysis results	
			*I* ^2^	*P*	RR, 95%CI	*P*
Incidence of grade I neurotoxicity	ADI + chemotherapy vs chemotherapy	1	NA	NA	0.79 (0.19, 3.30)	0.743
	SFI + chemotherapy vs chemotherapy	3	0%	0.998	0.73 (0.53, 1.00)	0.05
	SMI + chemotherapy vs chemotherapy	4	51.30%	0.104	0.72 (0.42, 1.26)	0.253
	SIFZI + chemotherapy vs chemotherapy	1	NA	NA	0.81 (0.44, 1.49)	0.5
	CKSI + chemotherapy vs chemotherapy	1	NA	NA	0.89 (0.37, 2.13)	0.791
	AI + chemotherapy vs chemotherapy	3	0%	0.935	**0.59 (0.36, 0.98)**	**0.042**
	KAI + chemotherapy vs chemotherapy	2	80.30%	0.024	0.67 (0.19, 2.36)	0.531
	EEI + chemotherapy vs chemotherapy	1	NA	NA	0.48 (0.10, 2.38)	0.369
	LI + chemotherapy vs chemotherapy	1	NA	NA	0.41 (0.16, 1.07)	0.069
	XAPI + chemotherapy vs chemotherapy	2	78.60%	0.031	0.43 (0.10, 1.78)	0.531
	BJOEI + chemotherapy vs chemotherapy	1	NA	NA	0.75 (0.18, 3.07)	0.689

Incidence of grade II neurotoxicity	ADI + chemotherapy vs chemotherapy	1	NA	NA	0.17 (0.02, 1.34)	0.099
	SFI + chemotherapy vs chemotherapy	3	0%	0.966	**0.43 (0.24, 0.79)**	**0.006**
	SMI + chemotherapy vs chemotherapy	4	33.90%	0.209	0.68 (0.43, 1.07)	0.095
	SIFZI + chemotherapy vs chemotherapy	1	NA	NA	0.77 (0.38, 1.570)	0.471
	CKSI + chemotherapy vs chemotherapy	1	NA	NA	0.86 (0.31, 2.38)	0.767
	AI + chemotherapy vs chemotherapy	3	0%	0.902	**0.39 (0.19, 0.81)**	**0.011**
	KAI + chemotherapy vs chemotherapy	1	NA	NA	0.17 (0.01, 3.40)	0.246
	EEI + chemotherapy vs chemotherapy	1	NA	NA	0.96 (0.15, 6.28)	0.966
	LI + chemotherapy vs chemotherapy	1	NA	NA	0.60 (0.11, 3.40)	0.566
	XAPI + chemotherapy vs chemotherapy	2	0%	0.328	0.54 (0.23, 1.31)	0.173
	BJOEI + chemotherapy vs chemotherapy	1	NA	NA	0.75 (0.18, 3.07)	0.689

Incidence of grade III neurotoxicity	ADI + chemotherapy vs chemotherapy	1	NA	NA	0.15 (0.01, 2.81)	0.204
	SFI + chemotherapy vs chemotherapy	3	0%	0.949	**0.16 (0.03, 0.87)**	**0.034**
	SMI + chemotherapy vs chemotherapy	4	7.40%	0.356	**0.38 (0.20, 0.75)**	**0.005**
	SIFZI + chemotherapy vs chemotherapy	1	NA	NA	0.25 (0.03, 2.15)	0.207
	CKSI + chemotherapy vs chemotherapy	1	NA	NA	1.00 (0.26, 3.79)	1
	AI + chemotherapy vs chemotherapy	3	0%	0.825	0.26 (0.07, 1.04)	0.056
	LI + chemotherapy vs chemotherapy	1	NA	NA	0.18 (0.01, 3.65)	0.265
	XAPI + chemotherapy vs chemotherapy	1	NA	NA	0.52 (0.09, 2.96)	0.462

Incidence of grade IV neurotoxicity	ADI + chemotherapy vs chemotherapy	1	NA	NA	0.35 (0.02, 8.34)	0.516
	SMI + chemotherapy vs chemotherapy	2	0%	0.718	0.51 (0.22, 1.22)	0.129
	AI + chemotherapy vs chemotherapy	1	NA	NA	0.33 (0.01, 8.00)	0.498

Incidence of grade I∼IV neurotoxicity	ADI + chemotherapy vs chemotherapy	10	13.50%	0.319	**0.42 (0.31, 0.57)**	**<0.0001**
	SFI + chemotherapy vs chemotherapy	3	0%	0.973	**0.57 (0.46, 0.71)**	**<0.0001**
	SMI + chemotherapy vs chemotherapy	4	67.80%	0.025	**0.56 (0.40, 0.78)**	**0.001**
	SIFZI + chemotherapy vs chemotherapy	2	0%	0.507	**0.68 (0.52, 0.89)**	**0.005**
	CKSI + chemotherapy vs chemotherapy	8	17.70%	0.29	**0.56 (0.45, 0.71)**	**<0.0001**
	HCSI + chemotherapy vs chemotherapy	2	0%	0.874	**0.45 (0.30, 0.69)**	**<0.0001**
	AI + chemotherapy vs chemotherapy	3	0%	0.997	**0.47 (0.33, 0.66)**	**<0.0001**
	KAI + chemotherapy vs chemotherapy	3	82.20%	0.004	0.45 (0.11, 1.79)	0.254
	KLEI + chemotherapy vs chemotherapy	1	NA	NA	**0.66 (0.45, 0.97)**	**0.034**
	EEI + chemotherapy vs chemotherapy	1	NA	NA	0.64 (0.21, 1.99)	0.441
	LI + chemotherapy vs chemotherapy	1	NA	NA	**0.40 (0.18, 0.85)**	**0.018**
	XAPI + chemotherapy vs chemotherapy	4	54.70%	0.085	**0.59 (0.38, 0.92)**	**0.019**
	BJOEI + chemotherapy vs chemotherapy	3	0%	0.809	0.84 (0.58, 1.21)	0.346

NA, data not available; RR, relative risk; CI, confidence interval; XAPI, Xiaoaiping injection; CKSI, compound kushen injection; ADI, Aidi injection; BJOEI, Brucea javanica oil emulsion injection; SMI, Shenmai injection; KAI, Kangai injection; AI, *Astragalus* injection; EEI, elemene emulsion injection; SFI, Shenfu injection; SIFZI, Shenqi Fuzheng injection; KLEI, Kanglaite injection; HCSI, Huachansu injection; LI, lentinan injection.

## Data Availability

The data used to support the findings of this study are available from the corresponding author upon request.

## Abbreviations

XAPI: Xiaoaiping injection
CKSI: Compound kushen injection
ADI: Aidi injection
BJOEI: Brucea javanica oil emulsion injection
SMI: Shenmai injection
KAI: Kangai injection
AI: *Astragalus* injection
EEI: Elemene emulsion injection
SFI: Shenfu injection
SIFZI: Shenqi Fuzheng injection
KLEI: Kanglaite injection
HCSI: Huachansu injection
LI: Lentinan injection
TCMIs: Traditional Chinese medicine injections
OIPN: Oxaliplatin-induced peripheral neurotoxicity
NMA: Network meta-analysis
RCTs: Randomized controlled trials
SUCRA: Surface under the cumulative ranking curve.

## Supplementary Materials

Supplementary Materials Table S1. Retrieval strategy of studies from the PubMed database. Figure S1. Sensitivity analysis of incidence of grade I neurotoxicity (SFI plus chemotherapy vs. chemotherapy). Figure S2. Sensitivity analysis of incidence of grade I neurotoxicity (SMI plus chemotherapy vs. chemotherapy). Figure S3. Sensitivity analysis of incidence of grade I neurotoxicity (AI plus chemotherapy vs. chemotherapy). Figure S4. Sensitivity analysis of incidence of grade II neurotoxicity (SFI plus chemotherapy vs. chemotherapy). Figure S5. Sensitivity analysis of incidence of grade II neurotoxicity (SMI plus chemotherapy vs. chemotherapy). Figure S6. Sensitivity analysis of incidence of grade II neurotoxicity (AI plus chemotherapy vs. chemotherapy). Figure S7. Sensitivity analysis of incidence of grade III neurotoxicity (SFI plus chemotherapy vs. chemotherapy). Figure S8. Sensitivity analysis of incidence of grade III neurotoxicity (SMI plus chemotherapy vs. chemotherapy). Figure S9. Sensitivity analysis of incidence of grade III neurotoxicity (AI plus chemotherapy vs. chemotherapy). Figure S10. Sensitivity analysis of incidence of grade III neurotoxicity (ADI plus chemotherapy vs. chemotherapy). Figure S11. Sensitivity analysis of incidence of grade I ∼ IV neurotoxicity (SFI plus chemotherapy vs. chemotherapy). Figure S12. Sensitivity analysis of incidence of grade I ∼ IV neurotoxicity (SMI plus chemotherapy vs. chemotherapy). Figure S13. Sensitivity analysis of incidence of grade I ∼ IV neurotoxicity (CKSI plus chemotherapy vs. chemotherapy). Figure S14. Sensitivity analysis of incidence of grade I ∼ IV neurotoxicity (AI plus chemotherapy vs. chemotherapy). Figure S15. Sensitivity analysis of incidence of grade I ∼ IV neurotoxicity (KAI plus chemotherapy vs. chemotherapy). Figure S16. Sensitivity analysis of incidence of grade I ∼ IV neurotoxicity (XAPI plus chemotherapy vs. chemotherapy). Figure S17. Sensitivity analysis of incidence of grade I ∼ IV neurotoxicity (BJOEI plus chemotherapy vs. chemotherapy).
